# Listening to self-chosen music regulates induced negative affect for both younger and older adults

**DOI:** 10.1371/journal.pone.0218017

**Published:** 2019-06-06

**Authors:** Jenny M. Groarke, Michael J. Hogan

**Affiliations:** 1 School of Psychology, Queen’s University Belfast, Belfast, United Kingdom; 2 School of Psychology, National University of Ireland, Galway, Galway, Ireland; University of Zurich, SWITZERLAND

## Abstract

The current study evaluated the efficacy of self-chosen music listening for the function of affect regulation comparing effects in younger and older adults. Forty younger (18–30 years, *M* = 19.75, *SD* = 2.57, 14 males) and forty older (60–81 years, *M* = 68.48, *SD* = 6.07, 21 males) adults visited the laboratory and were randomised to either the intervention (10 minutes of listening to self-chosen music) or the active control condition (10 minutes of listening to an experimenter-chosen radio documentary). Negative affect (NA) was induced in all participants using a speech preparation and mental arithmetic task, followed by the intervention/control condition. Measures of self-reported affect were taken at baseline, post-induction and post-intervention. Controlling for baseline affect and reactivity to the NA induction, in comparison with the active control group the music listening group demonstrated greater reduction in NA. Supporting developmental theories of positive ageing, analyses also found significant main effects for age, with older adults experiencing greater reduction of NA than younger adults, regardless of condition. Results of the current study provide preliminary insights into the effects of self-chosen music on induced NA, however, additional experimental control conditions comparing self-chosen and experimenter-chosen music with self-chosen and experimenter-chosen active controls are needed to fully understand music listening effects for affect regulation.

## Introduction

Music listeners highlight affect regulation as the most common and most important function of music [1]. Affect regulation functions of music listening have been associated with higher levels of wellbeing in survey studies [2]. Experimental studies of music listening have reported a range of effects that have implications for enhancing wellbeing, including increased positive affect and decreased negative affect [3], increased relaxation [4], and reduced stress [5]. However, fewer studies have examined the affect regulation effects of music listening following a stressor or negative affect induction. This type of study design provides a greater degree of experimental control by ensuring that participants in all conditions are experiencing a more similar affective experience at baseline, prior to the examination of music listening and control group effects.

The majority of younger and older adults listen to music daily [6, 7]. However, very little is known about potential differences in the effects of music listening on younger and older adults. Notably, when listening to music for the function of affect regulation, people select their own music [8]. However, there is the tendency for laboratory-based research to compare the effects of different types of experimenter-selected musical stimuli rather than allowing participants to select their own music to listen to, as they do in everyday life. Also, in everyday settings music listening is one activity amongst a range of activities that people might select to regulate negative affect. Experimental research in the area rarely compares music listening conditions with an active control, or a non-musical control condition. To gain a greater understanding of the link between music listening and wellbeing in everyday life amongst younger and older adults there is a need for more ecologically valid and well-controlled experimental studies examining the effects of self-selected music for the function of affect regulation. The current study sought to advance understanding of everyday music listening and build upon previous experimental research by examining the affect regulating effects of a self-chosen music listening intervention following a stress induction for both younger and older adults.

### The effects of music on younger and older adults

Carstensen’s Socio-Emotional Selectivity Theory [9] proposes three motives that moderate age-related changes in social interaction: emotion regulation, identity development, and information seeking. As adults advance into old age, it is theorised that a limited chronological future directs attention toward the affective dimension of social interaction. This shift in socio-emotional goals brought about by ageing may enhance certain aspects of emotional functioning and the regulation of affective experience. For example, a number of empirical studies have found that older adults report a lower experience of NA and fewer fluctuations in mood compared with younger adults, suggesting better regulation and optimisation of affective experience [10,11]. It is possible these developmental changes in affect regulation influence the effects of music listening in everyday life for older people.

According to socio-emotional selectivity theory older adults prioritise emotion regulation so that less emphasis is placed on negative information than positive information. The positivity effect is a bias in older adults’ attention towards positively-valenced stimuli. This positivity bias is observed in a variety of perceptual and memory tasks [12] and is also observed in older adults’ responses to music on outcomes other than affect regulation. Specifically, older adults demonstrate enhanced recognition of positive versus negative affect in musical stimuli, and increased emotional response to music expressing positive affect and reduced response to negative affect in music [13–15]. The current study extends previous research on age differences in perception and recognition of emotions in music by examining, for the first time, the impact of age on affect regulation by music listening. Although findings consistent with the tenets of socio-emotional selectivity theory have been demonstrated across a variety of experiences and experimental paradigms, research is needed on the effect of age on musical affect regulation to further evaluate the generalisability of the theory.

Previous survey research demonstrates age differences in the prevalence of use of discrete functions of music listening, with older adults reporting using music for affective reasons less frequently than younger adults [16, 17]. These results might imply that older adults have less need to use music for affect regulation because of developmental improvements in affective functioning described above. As such, a number of possibilities arise: older adults may be less practiced, and potentially less skilled in using music for affect regulation. In this situation one would predict reduced efficacy of music for regulating induced NA in the current study. Alternatively, older adults’ improved regulation ability may potentiate the regulating effects of music. Examining differences in the response to music between younger and older adults increases our understanding of the effects of music across the lifespan. If effects are similar in both age groups this may suggest affect regulation effects of music are both adaptive and stable across the lifespan. Studies in music psychology have predominately employed samples of young people. Including older adult participants therefore may broaden our knowledge of the effect of music on wellbeing. Further, contrasts in effects of music between younger and older adults may provide theoretical insight into the distinct ways people regulate affect at different stages of life.

#### Selecting control conditions

When examining the effects of music listening Chanda and Levitin [18] recommend the use of active control conditions that may provide similar rewards as music in terms of level of arousal, attentional capture, and affective engagement. They make more specific recommendations to compare music listening with similarly rewarding leisure activities like watching television or reading. As the current study is focused on music listening, it was decided that an auditory active control condition would be appropriate, specifically, listening to an engaging radio documentary.

A small number of studies have compared music listening to alternative experimental conditions where participants are engaged in an activity. Scheufele [19] compared the effectiveness of two relaxation interventions (progressive relaxation techniques and classical music), with both an active control (i.e., completing memory tasks) and a silent control condition for reducing induced stress. While some studies found music listening to be more effective for sadness regulation than either writing or solving geometry problems [20,21], Scheufele [19] concluded that progressive relaxation was the more effective tactic for affect regulation.

Silence is the most common control condition in studies of music listening. One problem is that sitting in silence completely unoccupied is a unique and somewhat infrequent scenario. Greater certainty regarding the efficacy of music listening for affect regulation would be gained from comparing listening to music with other activities that may support affect regulation in everyday contexts. If significant effects of music are found against an active control this may be considered a strong and ecologically valid endorsement of the benefits of music, as everyday responses to stress may involve a choice between listening to music versus other forms of everyday activity.

#### Selecting music

The vast majority of studies have prescribed classical, or ‘relaxing’ music [22], but there are studies that have had participants choose their own musical stimuli, or choose from the experimenter’s selection [23–29]. These studies advance upon existing designs as they more closely resemble everyday music listening contexts where listeners select their own music for affect regulation.

Of the studies incorporating self-selected music, only the studies by Labbé et al. [24], Radstaak et al. [25] and Burns et al. [26] examined self-chosen music following a stressor, and found that even in the context of ongoing NA, music chosen by participants for the function of relaxation has better, or at the very least, similar affect regulation effects relative to experimenter-chosen music.

Music chosen by participants is likely to vary in terms of its valence and arousal, as well as other musical properties known to predict affective responses to music [30]. When participant-selected music is used researchers may gain increased ecological validity, but relinquish a degree of experimental control over the intervention stimulus. At the same time, previous experimental research has found that people have stronger and more positive responses to music which is self-chosen, even to high-arousal and negatively-valenced music [29], possibly due to increased preference, familiarity, and memories associated with the music [31, 32]. Thus, self-chosen music may be more suitable than experimenter-chosen music, especially if a stress or NA induction is employed.

### The current study

The current study is the first RCT of music and affect regulation to compare effects in younger and older listeners. Furthermore, it is the first study to examine the affect regulation effects of music with healthy older adults incorporating both a stressor and an active control condition. Drawing upon research focused on affect regulation effects of self-chosen music listening, the limited body of empirical music psychology research with older adults, and theoretical and empirical work on lifespan changes in affective functioning, a number of hypotheses are proposed:

First, it is hypothesised that, when compared with an active control, a self-chosen music listening intervention will be more effective in reducing induced NA in both younger and older adults [25, 26]. Second, consistent with research [10, 11], older adults will report less negative affect at baseline than younger adults. Third, given research findings suggesting that older adults have greater affect regulation abilities than younger adults [9, 10], it is hypothesised that older adults will experience greater regulation of NA than younger adults in both experimental conditions.

## Materials and methods

This study was conducted according to the principles expressed in the Declaration of Helsinki. All procedures were reviewed and approved by the Research Ethics Committee at NUI, Galway (Ref: 15/JAN/08).

### Participants

Participants were recruited via advertisements in local and national media seeking volunteers to complete a questionnaire on music listening, and furthermore to take part in a laboratory session examining the effect of music on task performance. Undergraduate students were also recruited through an online research participation system and received course credits for their participation, whilst travel costs and expenses were covered as an incentive for the older participants. Participants were included if they reported having normal or corrected-to-normal hearing, and spoke competent English, and excluded if they were taking sedative medication, were drug or alcohol dependent, or had a psychological condition or affective disorder (i.e., depression, PTSD, generalised anxiety disorder, social anxiety).

Data were collected from 40 younger adults (YA) between the ages of 18 and 30 (*M* = 19.75, *SD* = 2.57, 14 males), and 40 older adults between the ages of 60 and 81 years old (*M* = 68.48, *SD* = 6.07, 21 males). Table 1 presents demographic data for younger and older adults.

**Table 1:** Demographic characteristics of younger and older adult participants.

	Younger Adults	Older Adults
**Highest Level of Education**		
Postgraduate	2.5%	35%
Undergraduate	7.5%	40%
Secondary School	90%	10%
Primary School		15%
**Working**	17.5%	17.5%
Retired		67.5%
Self-employed		2.5%
Homemaker		5%
Unemployed		7.5%
**Nationality**		
Ireland	80%	92.5%
United Kingdom	7.5%	5%
United States	10%	
Latvia	2.5%	
New Zealand		2.5%

### Materials

#### Demographic questions

These assessed age, gender, nationality, highest level of education achieved, employment status, and whether participants were currently students.

#### Music choices

Participants were asked to provide the artist, song title, and genre of 15 minutes worth of music that they would listen to in a stressful situation, and fifteen minutes worth of music that they would listen to in a social situation.

#### The adaptive functions of music listening scale [4]

Participants rate their level of agreement with 46 items representing efficacy beliefs and outcome expectations of a range of music listening functions using a 5-point Likert scale ranging from 1 (strongly disagree) to 5 (strongly agree).

**Subjective affect** was measured using Visual Analogue Scales (VAS), on which participants indicate how they are feeling in the moment on a scale from 0–10 between two bipolar affective states. The VAS includes 8 items: 1. Alert-Bored, 2. Excited-Depressed, 3. Happy-Sad, 4. Calm-Tense, 5. Content-Upset, 6. Relaxed-Nervous, 7. Active-Fatigue, and 8. Not Stressed-Stressed. Higher scores are indicative of more NA. These target affects were drawn from Russell’s circumplex model of affect [33].

**The trier social stress test (TSST)** [34] was used to induce mild-moderate psychosocial stress in all participants. In the standard TSST procedure participants are told to imagine they are invited to a job interview. There are three phases: 1) a speech preparation phase; 2) a free-speech phase where they describe why they are the best candidate for the job; 3) a mental arithmetic task. Aspects of the TSST can be modified to meet the needs of different research programs [35]. In the current study, the TSST was applied without the free-speech phase. Participants had a five-minute speech preparation phase, and were then told that they will give the speech at the end of the laboratory session and that it would be recorded for subsequent voice and behavioural analysis. Next the participant performed an arithmetic task, counting backwards from 2023 in steps of 17 aloud for five minutes—having to restart every time an error was made. Due to the focus on subjective affect in the current study, a waiting period of 10 minutes prior to the baseline assessment was deemed sufficient for habituation [34]. All phases of the TSST were carried out in the presence of two female experimenters.

#### Familiarity and perceived efficacy of music selected for stressful situation

During debriefing participants were asked to rate their music playlists on a 5-point scale from 0 (not at all familiar) to 4 (extremely familiar). Participants were also asked to rate how effective they believed that music to be for regulating negative feelings—such as those aroused by the stress induction on a scale of 0 (not at all) to 4 (extremely).

#### Audio

Audio was played via PC through SONY over-ear headphones. Each participant selected a volume that was comfortable for them for the auditory stimulus.

### Procedure

After initial contact (by email, phone or online recruitment system) and participant screening for inclusion criteria, all eligible participants were mailed or emailed the Participant Information Sheet. At this stage, participants answered demographic questions, made music choices for a stressful situation, and a social situation, and completed the Adaptive Functions of Music Listening Scale. Using a random number generator, twenty younger adult participants (6 males) and twenty older adults (12 males) were randomised to the intervention. Twenty younger (8 males) and twenty older adults (9 males) were also randomised to the control arm. Condition allocation was concealed from participants until debriefing.

**Fig 1:**
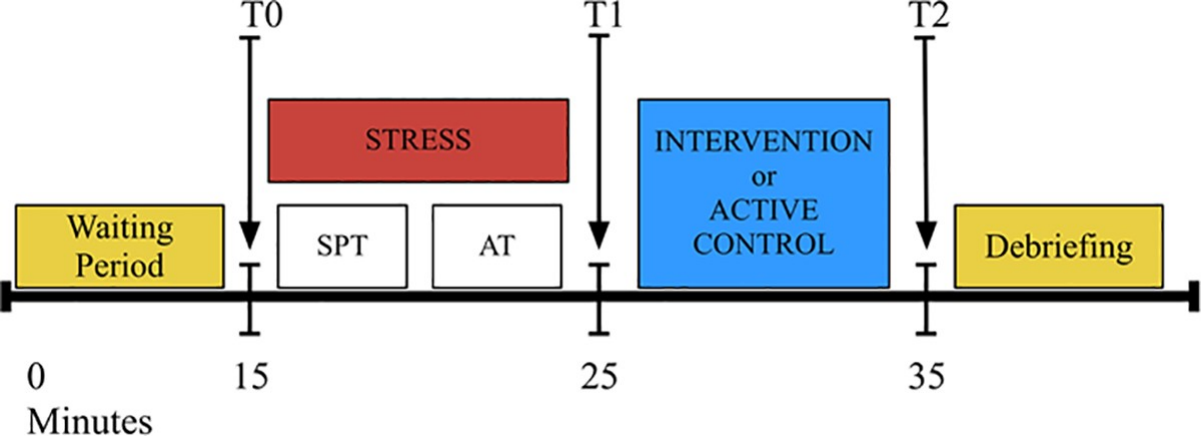
Experimental procedure. **Notes;** SPT = Speech preparation task, AT = Arithmetic task, T0 = baseline assessment of NA, T1 = post-induction assessment, T2 post-intervention assessment, Intervention = listening to self-chosen music, Active control = Listening to a radio documentary.

In the laboratory session, after providing written consent, participants rated their baseline level of affect on each of the 8 VAS. Next, NA was induced in all participants using the TSST (described above). Participants rated their affect on the VAS post-induction. Participants in the intervention condition then listened to the first 10 minutes of music from their selection of music chosen for a stressful situation. Participants in the control condition listened to a 10-minute radio documentary on Charles Darwin. All participants were told that this 10-minute break was a rest period during which the experimenters would prepare the audio-visual room for their speech. Finally, affect was self-rated post-intervention (Fig 1). Participants in the control condition listened to their self-selected music following the final assessment. Participants were debriefed, and told they would not be making the speech. The deception regarding the stress induction and nature of the research question were revealed and retrospective written consent was sought. All participants were asked to rate the familiarity of the music they selected for stressful situations, as well as their rating of how effective they believe that music to be for regulating NA.

#### Power

The statistical program G*Power was used to conduct power analysis. Adhering to Cohen's [36] guidelines for small (r = 0.1), medium (r = 0.3), and large (r = 0.5) effects, two-tailed alpha of .05 was assumed for all tests. With 4 groups (younger adult intervention, younger adult control, older adult intervention, older adult control), 2 treatments (intervention, control), 2 covariates (baseline affect, affect reactivity), as well as a medium effect size observed in previous research [24, 26, 37] and a power of 0.80, the recommended total sample size for ANCOVA was 79.

## Results

A large number of statistical tests are reported below. There is increased risk of Type I errors (finding a false positive) when testing multiple hypotheses, using multiple tests, and multiple outcome measures. P-value adjustments (e.g., the Bonferroni correction) reduce the chance of making Type I errors, but increase the chance of making Type II errors (finding a false negative) [38]. In the current study statistical significance was determined as *p* < .05. However, when interpreting the findings, the reader should balance the reported significance level with the magnitude of effect, the quality of the study, and with findings of other studies.

Twenty-five different genres of music were selected by participants for a stressful situation (see Table 2). More than one third of the songs selected by older adults for affect regulation were classical pieces (37%), whereas less than 3% of the songs chosen by younger adults were classical. Further, 66% of older adults selected at least one classical music piece, compared to 5% of younger adults. One third of the younger adult music choices, however, were pop songs, compared to only 16% of the older adults’ selections.

**Table 2:** Genres of music selected for the function of affect regulation by 40 older adults and 40 younger adults.

	Older Adults		Younger Adults	
Total number of musical pieces selected:	140		173	
**Classical**	52	37.14%	5	2.89%
**Choral & Sacred Chant**	10	7.14%		
**Opera**	7	5.00%		
**Instrumental**	2	1.43%	1	0.58%
**Pop**	23	16.43%	64	36.99%
**Country**	12	8.57%	5	2.89%
**Folk**	4	2.86%	16	9.25%
**Jazz/Swing**	7	5.00%	2	1.16%
**Motown/Soul**	2	1.43%	1	0.58%
**Blues**	1	0.71%		
**Rock**	8	5.71%	13	7.51%
**Indie**			24	13.87%
**Metal**			3	1.73%
**Alternative**			3	1.73%
**Punk**			7	4.05%
**Easy listening**	1	0.71%		
**Musical theatre**	1	0.71%		
**Traditional Irish**	10	7.14%		
**Electronic/Ambient**			6	3.27%
**Drum & Bass/Dubstep**			3	1.73%
**Techno/Dance**			5	2.89%
**Rap**			5	2.89%
**Hip Hop**			4	2.31%
**R&B**			2	1.16%
**Trap**			4	2.31%

At the debriefing stage, all participants were asked to rate the musical stimuli by familiarity and perceived affect-regulation efficacy. 60% of participants rated their music playlists as extremely familiar, 30% as moderately familiar, and 10% selected music that was somewhat familiar. Participants were also asked to rate their music choices in terms of perceived efficacy. 45% of participants rated their music choices as extremely effective, 25% as moderately effective, 22.5% rated it as somewhat effective, and 7.5% rated their music as not at all effective for regulating negative affect. Independent t-tests demonstrated that Familiarity or Perceived Efficacy of music scores did not differ by condition (*Familiarity*: t(78) = −1.05, *p* = .30; *Efficacy*: t(78) = −1.11, *p* = .27) or by age group (*Familiarity*: t(78) = 1.07, *p* = .29; *Efficacy*: t(78) = −1.24, *p* = .22).

To assess the need to incorporate covariates into the main analyses, the intervention and control group, and younger and older adult groups were compared with each other on VAS at baseline. Unexpectedly, the control group were significantly more *Stressed* at baseline than the intervention group. Consistent with expectations, older adults were significantly less *Sad*, *Upset*, *Bored*, *Fatigued*, and *Stressed* than younger adults at baseline (see Table 3). Baseline NA was included as a covariate in each analysis.

**Table 3:** Independent samples t-tests comparing the control and intervention group, and the younger and older adult samples on their baseline level of self-reported NA by VAS.

Baseline NA	Control n = 40 M*(SD)*	Intervention n = 40 M*(SD)*	95% CI mean difference	t(78)	*p*	*d*	Younger adults n = 40 M*(SD)*	Older adults n = 40 M*(SD)*	95% CI mean difference	t(78)	*p*	*d*
**Stress**	2.97*(1*.*93)*	2.00*(1*.*66)*	0.17, 1.77	2.41	0.02	0.54	3.03*(1*.*89)*	1.94*(1*.*68)*	0.29, 1.87	2.71	0.002	0.61
**Nervousness**	2.18*(1*.*96)*	2.25*(1*.*66)*	-0.88, 0.74	-0.16	0.87		2.50*(1*.*81)*	1.93*(1*.*78)*	-0.23, 1.36	1.41	0.16	0.31
**Tension**	2.44*(2*.*08)*	2.38*(1*.*75)*	-0.79, 0.92	0.16	0.88		2.45*(1*.*75)*	2.37*(2*.*08)*	-0.77, 0.94	0.19	0.85	
**Upset**	1.72*(1*.*48)*	1.40*(1*.*26)*	-0.30, 0.93	1.03	0.31		2.00*(1*.*32)*	1.11*(1*.*30)*	0.30, 1.47	3.01	0.003	0.67
**Sadness**	2.49*(1*.*67)*	2.35*(1*.*78)*	-0.62, 0.91	0.37	0.71		2.93*(1*.*25)*	1.92*(1*.*97)*	0.27, 1.74	2.72	0.008	0.61
**Depressed Affect**	2.82*(1*.*50)*	2.85*(1*.*56)*	-0.71, 0.65	-0.08	0.94		2.88*(1*.*38)*	2.80*(1*.*67)*	-0.60, 0.76	0.23	0.82	
**Fatigue**	3.48*(1*.*91)*	3.34*(1*.*98)*	-0.73, 1.00	0.31	0.76		4.10*(1*.*82)*	2.73*(1*.*81)*	0.56, 2.18	3.36	0.001	0.75
**Boredom**	2.55*(1*.*79)*	2.38*(2*.*27)*	-0.74, 1.09	0.38	0.71		3.15*(1*.*74)*	1.77*(2*.*09)*	0.52, 2.23	3.19	0.008	0.72

*Notes; d* = Cohen’s *d* estimate of effect size; .20 (small); .50 (medium); .80 (large).

### Efficacy of NA induction

A series of paired-samples t-tests were conducted to evaluate the impact of the TSST on participants’ level of self-reported NA post-induction. There was a statistically significant increase in *Stress*, *Nervousness*, *Tension*, *Upset*, *Sadness*, and *Depressed* affect from baseline to post-induction. There was no statistically significant increase in *Boredom* or *Fatigue*, thus they will not be included in further analyses (See Table 4). The difference between self-reported NA at baseline (pre-induction) and post-induction (Time 2) was calculated for each VAS to determine participants’ level of reactivity to the induction. A higher score indicates more reactivity, or a higher degree of NA induction by the TSST. A series of two-way ANOVA’s (see Table 5) confirmed that NA Reactivity score did not significantly differ by condition. NA Reactivity did not differ by age group, contrary to predictions.

**Table 4:** Descriptive statistics for 80 participants baseline affect (Time 1) and post-induction affect (Time 2), and results of paired samples t-tests demonstrating efficacy of na induction.

VAS	Time 1 NA M*(SD)*	Time 2 NA M*(SD)*	95% CI mean difference	t(79)	*p*	*d*
**Stress**	2.49*(1*.*85)*	4.76*(2*.*52)*	-2.82, -1.74	-8.36	< 0.001	0.93
**Nervousness**	2.22*(1*.*81)*	4.68*(2*.*17)*	-3.01, -1.90	-8.80	< 0.001	0.98
**Tension**	2.41*(1*.*91)*	4.98*(2*.*42)*	-3.24, -1.90	-7.61	< 0.001	0.85
**Upset**	1.56*(1*.*38)*	3.48*(2*.*09)*	-2.37, -1.46	-8.34	< 0.001	0.93
**Sadness**	2.42*(1*.*72)*	3.25*(1*.*95)*	-1.26, -0.40	-2.27	< 0.001	0.25
**Depressed Affect**	2.84*(1*.*52)*	3.30*(1*.*90)*	-0.87, -0.06	-2.27	0.03	0.25
**Fatigue**	3.42*(1*.*93)*	3.53*(2*.*44)*	-0.69, 0.47	-0.37	0.71	
**Boredom**	2.46*(2*.*04)*	2.18*(1*.*70)*	-0.14, 0.72	1.33	0.19	

*Notes*; d = Cohen’s d estimate of effect size; .20 (small); .50 (medium); .80 (large)

**Table 5:** Results of 2x2 ANOVA demonstrating no significant group or age differences in reactivity to a NA induction.

	Group	Age	Group x Age	Intervention	Control	Younger Adults	Older Adults
DV; NA Reactivity Score	*F*_(1,76)_ *(p)*	*F*_(1,76)_ *(p)*	*F*_(1,76)_ *(p)*	M*(SD)*	M*(SD)*	M*(SD)*	M*(SD)*
**Stress Reactivity**	2.77 *(0*.*10)*	1.97 *(0*.*16)*	0.30 *(0*.*59)*	2.73*(2*.*37)*	1.83*(2*.*44)*	1.90*(1*.*88)*	2.65*(2*.*86)*
**Nervous Reactivity**	3.13 *(0*.*08)*	0.56 *(0*.*46)*	0.11 *(0*.*74)*	2.95*(2*.*55)*	1.97*(2*.*38)*	2.25*(2*.*35)*	2.67*(2*.*65)*
**Tension Reactivity**	0.82 *(0*.*37)*	0.12 *(0*.*73)*	0.50 *(0*.*48)*	2.88*(2*.*78)*	2.26*(3*.*23)*	2.45*(2*.*82)*	2.68*(3*.*23)*
**Upset Reactivity**	1.52 *(0*.*22)*	1.61 *(0*.*21)*	0.00 *(0*.*97)*	2.20*(1*.*77)*	1.63*(2*.*29)*	1.63*(1*.*75)*	2.21*(2*.*31)*
**Sadness Reactivity**	0.11 *(0*.*74)*	0.86 *(0*.*36)*	0.22 *(0*.*64)*	0.90*(1*.*88)*	0.76*(2*.*00)*	0.63*(1*.*67)*	1.03*(2*.*14)*
**Depressed Affect Reactivity**	0.19 *(0*.*67)*	2.31 *(0*.*13)*	0.10 *(0*.*76)*	0.38*(1*.*60)*	0.55*(2*.*05)*	0.78*(1*.*48)*	0.15*(2*.*10)*

### Efficacy of music listening intervention

A series of six 2x2 between-groups ANCOVA were conducted to assess the effectiveness of a music listening intervention in regulating induced negative affect relative to an active control for younger and older adults. The IVs were condition (music listening intervention, active control) and age group (younger, older adults). The DV was the NA Regulation Score for self-reported 1. *Stress*, 2. *Nervousness*, 3. *Tension*, 4. *Upset*, 5. *Sadness*, and 6. *Depressed Affect*. These scores were calculated as the difference between self-reported level of NA at Time 3 (post-intervention) and Time 2 (post-induction), and therefore reflect the degree or extent of regulation achieved. Higher scores represent greater regulation of, or more recovery from, induced NA. Self-reported NA at Baseline (pre-induction), and NA Reactivity score were used as covariates to control for group and individual differences. The results of each of these analyses are described below and summarised in Table 6. The means and SD for NA reported at each timepoint for both conditions and both age groups are presented in Table 7. Given multiple comparisons a more conservative alpha level of 0.01 may be preferred for interpreting mean differences in light of the Bonferroni approach.

**Table 6:** Results of a 2x2 Between Subject ANCOVA evaluating the efficacy of a music listening intervention versus active control for 40 younger adults and 40 older adults.

	Group X Age	Group		Age		Intervention		Control		Younger Adults		Older Adults	
DV; NA Regulation Score	*F*_(1,74)_	*F*_(1,74)_	η_p_^2^	*F*_(1,74)_	η_p_^2^	*M* (SD)	*AdjM* (SE)	*M* (SD)	*AdjM* (SE)	*M* (SD)	*AdjM* (SE)	*M* (SD)	*AdjM* (SE)
**Stress Regulation**	1.02	5.24*	.07	7.61**	.09	2.63 (2.51)	2.49 (.25)	1.58 (2.31)	1.72 (.25)	1.58 (2.07)	1.63 (.25)	2.63 (2.71)	2.57 (.26)
**Nervous Regulation**	2.09	15.37***	.17	8.57**	.10	3.20 (2.34)	2.84 (.23)	1.18 (1.85)	1.54 (.23)	1.70 (1.80)	1.71 (.23)	2.68 (2.70)	2.66 (.23)
**Tension Regulation**	1.33	3.22		1.45		3.20 (2.68)	2.95 (.25)	2.08 (2.61)	2.32 (.25)	2.35 (2.26)	2.43 (.25)	2.93 (3.06)	2.85 (.25)
**Upset Regulation**	2.13	5.08*	.06	4.54*	.06	1.58 (2.27)	1.37 (.23)	0.43 (2.33)	0.64 (.23)	0.50 (2.16)	0.64 (.23)	1.50 (2.47)	1.36 (.23)
**Sadness Regulation**	0.11	6.64**	.08	6.99**	.09	1.05 (2.32)	1.02 (.25)	0.08 (2.02)	0.10 (.25)	0.13 (2.16)	0.07 (.26)	1.00 (2.21)	1.58 (.37)
**Depressed Affect Regulation**	0.03	6.14*	.08	25.50***	.26	0.23 (1.53)	0.28 (.21)	-0.40 (1.97)	-0.45 (.21)	-0.63 (1.88)	-0.84 (.21)	0.45 (1.52)	0.67 (.21)

Notes: AdjM = Adjusted Mean, SE = Standard Error, η_p_^2^ = Partial Eta Squared .01 (small); .06 (medium); .14 (large)

* *p* < .05

** *p* < .01

*** *p* < .001. Exact p-values are presented in text above.

**Table 7:** Means and SD for the control and intervention group, and the younger and older adult samples on level of self-reported NA measured at three timepoints.

	Control	Intervention	Younger adults	Older adults
DV	M*(SD)*	M*(SD)*	M*(SD)*	M*(SD)*
**Stress**				
Baseline	2.97*(1*.*93)*	2.00*(1*.*66)*	3.02*(1*.*89)*	1.94*(1*.*67)*
Post-induction	4.80*(2*.*50)*	4.72*(2*.*57)*	4.92*(2*.*15)*	4.60*(2*.*86)*
Post-intervention	3.23*(1*.*93)*	2.10*(1*.*72)*	3.35*(1*.*95)*	1.97*(1*.*57)*
**Nervousness**				
Baseline	2.18*(1*.*96)*	2.25*(1*.*66)*	2.50*(1*.*81)*	1.93*(1*.*78)*
Post-induction	4.15*(2*.*07)*	5.20*(2*.*16)*	4.75*(1*.*89)*	4.60*(2*.*44)*
Post-intervention	2.97*(1*.*85)*	2.00*(1*.*57)*	3.05*(1*.*89)*	1.92*(1*.*45)*
**Tension**				
Baseline	2.44*(2*.*08)*	2.37*(1*.*75)*	2.45*(1*.*75)*	1.92*(1*.*97)*
Post-induction	4.70*(2*.*44)*	5.25*(2*.*40)*	4.90*(2*.*10)*	2.95*(2*.*09)*
Post-intervention	2.62*(1*.*64)*	2.05*(1*.*74)*	2.55*(1*.*69)*	1.95*(1*.*92)*
**Upset**				
Baseline	1.72*(1*.*48)*	1.40*(1*.*26)*	2.00*(1*.*32)*	1.12*(1*.*30)*
Post-induction	3.35*(2*.*13)*	3.60*(2*.*07)*	3.62*(1*.*82)*	3.32*(2*.*35)*
Post-intervention	2.92*(1*.*77)*	2.02*(1*.*85)*	3.12*(1*.*88)*	1.82*(1*.*59)*
**Sadness**				
Baseline	2.49*(1*.*67)*	2.35*(1*.*77)*	2.92*(1*.*25)*	1.92*(1*.*97)*
Post-induction	3.25*(2*.*01)*	3.25*(1*.*90)*	3.55*(1*.*77)*	2.95*(2*.*09)*
Post-intervention	3.17*(1*.*74)*	2.20*(2*.*08)*	3.42*(1*.*74)*	1.95*(1*.*92)*
**Depressed Affect**				
Baseline	2.82*(1*.*50)*	2.85*(1*.*56)*	2.87*(1*.*38)*	2.80*(1*.*67)*
Post-induction	3.38*(2*.*03)*	3.22*(1*.*77)*	3.65*(1*.*73)*	2.95*(2*.*01)*
Post-intervention	3.77*(1*.*67)*	3.00*(1*.*87)*	4.27*(1*.*50)*	2.50*(1*.*65)*

#### 1. Stress Regulation

After adjusting for baseline *Stress* and *stress reactivity* to the induction, there was no significant group x age interaction effect for *Stress Regulation* (*p* = .32). There was a significant main effect of group (*p* = .02) and of age (*p* = .007). Inspection of means in Table 4 shows that those in the music listening intervention experienced significantly greater regulation of *Stress* than those in the control group. Older adults reported greater *Stress Regulation* than younger adults overall.

#### 2. Nervous Regulation

There was no significant group x age interaction effect for *Nervous Regulation* (*p* = .15). There was a significant main effect of group (*p* < .001) and of age (*p* = .005). Those in the intervention experienced significantly greater regulation of *Nervousness* than those in the control group, and older adults reported greater *Nervous Regulation* than younger adults.

#### 3. Tension Regulation

There was no significant group x time interaction (*p* = .25), and no main effects for group (*p* = .08), or age (*p* = .23) for *Tension Regulation*.

#### 4. Upset Regulation

There was no significant group x age interaction effect found (*p* = .149). There was a significant main effect of group (*p* = .03) and of age (*p* = .01). Those in the music listening intervention experienced significantly greater regulation of *Upset* than those in the control group. Older adults reported greater *Upset Regulation* than younger adults overall.

#### 5. Sadness Regulation

No interaction effect between group and age was found (*p* = .74). There was a main effect for group (*p* = .01), with the intervention group reporting significantly greater *Sadness Regulation* than participants in the control group. A main effect for age was also found (*p* = .01), with older adults reporting greater regulation of *Sadness* than younger adults.

#### 6. Depressed Affect Regulation

No significant group x age interaction effect was found for the regulation of *Depressed Affec*t (*p* = .86). There was a significant main effect of group (*p* = .02) and of age (*p* < .001). Those in the music listening intervention experienced significantly greater regulation of *Depressed Affect* than those in the control group. Older adults reported greater *Depressed Affect Regulation* than younger adults overall.

The hypothesis that a self-chosen music listening intervention would be more effective than an active control in regulating induced NA was supported. As predicted, older adults experienced greater regulation of NA than younger adults in both conditions.

### Additional analyses

#### Predictive effect of music listening beliefs

A series of regression analyses were conducted to address whether beliefs regarding perceived regulatory effects of music in everyday contexts, as measured by the Adaptive Functions of Music Listening scale, predict regulatory effects of music in the current RCT. Based on the results of correlation analyses in Groarke and Hogan [4], it was predicted that higher scores on the Anxiety Regulation subscale would predict higher levels of affect regulation, and that higher scores on the Rumination subscale would predict lower levels of affect regulation in the current RCT. A second series of regression analyses were carried out to examine the predictive effect of listeners’ beliefs about the specific music chosen and heard during the intervention. Social Cognitive Theory states that previous experiences influence beliefs and expectations in regard to the effects of future behaviour [39] and previous research in music psychology suggests that past functional success with music influences the effects that are experienced [40]. Therefore, it was expected that participants’ perceived efficacy of their selected music for the function of affect regulation would predict greater regulation of NA. Also, given that familiarity of music is associated with stronger emotional responding [31, 32], it was predicted that that higher ratings of familiarity would predict greater regulation of NA.

The regression analyses focused on the 40 participants in the intervention group (20 younger adults, 20 older adults). The DV was the NA Regulation score (1. *Stress Regulation*, 2. *Nervous Regulation*, 3. *Upset Regulation*, 4. *Sadness Regulation*, 5. *Depressed Affect Regulation*). This score measures the reduction in NA experienced by participants from pre to post music listening. Means and standard deviations for each variable included in regression analyses are presented in Table 8.

**Table 8:** Descriptive statistics for all study variables for 40 participants included in regression analyses.

	Younger Adults (n = 20) M*(SD)*	Older Adults (n = 20) M*(SD)*
**IV**		
**Anxiety Regulation**	3.73*(0*.*82)*	3.78*(0*.*63)*
**Rumination**	3.10*(0*.*84)*	2.56*(1*.*24)*
**Baseline Stress**	2.50*(2*.*01)*	1.50*(1*.*05)*
**Baseline Nervous**	2.30*(1*.*66)*	2.20*(1*.*70)*
**Baseline Upset**	1.90*(1*.*37)*	0.900*(*.*91)*
**Baseline Sadness**	3.10*(1*.*29)*	1.60*(1*.*90)*
**Baseline Depressed Affect**	3.10*(1*.*41)*	2.60*(1*.*70)*
**Stress Reactivity**	2.20*(1*.*85)*	3.25*(2*.*75)*
**Nervous Reactivity**	2.65*(2*.*34)*	3.25*(2*.*77)*
**Upset Reactivity**	1.90*(1*.*45)*	2.50*(2*.*03)*
**Sadness Reactivity**	0.80*(1*.*40)*	1.00*(2*.*29)*
**Depressed Affect Reactivity**	0.75*(1*.*29)*	0.00*(1*.*81)*
**Familiarity**	3.81*(0*.*42)*	3.73*(0*.*55)*
**Perceived Efficacy**	2.82*(0*.*82)*	3.51*(0*.*85)*
**DV**		
**Stress Regulation**	1.80*(2*.*14)*	3.45*(2*.*62)*
**Nervous Regulation**	2.30*(1*.*56)*	4.10*(2*.*67)*
**Upset Regulation**	0.85*(2*.*08)*	2.30*(2*.*27)*
**Sadness Regulation**	0.75*(2*.*12)*	1.35*(2*.*52)*
**Depressed Affect Regulation**	-0.20*(1*.*61)*	0.65*(1*.*35)*

#### Predictive effect of the Adaptive Functions of Music Listening Scale

Scores on the Anxiety Regulation and Rumination subscales of the AFML scale were entered as IVs in the first step. Participants’ Baseline NA and NA Reactivity were entered as IVs in Step 2 of the regression analyses. Results are presented in Table 9.

**Table 9:** Hierarchical multiple regression examining the predictive effect of the adaptive functions of music listening scale in 20 younger and 20 older adults assigned to a self-chosen music listening intervention.

	Stress Regulation			Nervous Regulation			Upset Regulation			Sadness Regulation			Depressed affect Regulation		
	F_(4,35)_ = 22.73, p < .001, R^2^ = .72, Adj R^2^ = .69			F_(4,35)_ = 13.81, p < .001, R^2^ = .61, Adj R^2^ = .57			F_(4,35)_ = 11.94, p < .001, R^2^ = .58, Adj R^2^ = .53			F_(4,35)_ = 10.70, p < .001, R^2^ = .55, Adj R^2^ = .50			F_(4,35)_ = 2.16, p = .05, R^2^ = .20, Adj R^2^ = .11		
Predictors	β	Fch	R^2^ ch	β	Fch	R^2^ ch	β	Fch	R^2^ ch	β	Fch	R^2^ ch	β	Fch	R^2^ ch
**Step 1**		3.23*	.10		2.80	.09		0.87	-.01		0.56	-.02		1.01	.00
**(i)** **Anxiety Regulation**	-.22			.02			.10			.04			.12		
**(ii) Rumination**	-.41*			-.36*			-.15			-.15			-.16		
**Step 2**		36.09***	.59		21.69***	.48		22.02***	.54		20.27***	.48		3.19*	.11
**(i)** **Baseline NA**	.31**			.50***			.10			.19			.28		
**(ii)** **NA Reactivity**	.78***			.86***			.74***			.79***			.38*		

Notes

* p < .05

** p < .01

*** p < .001.

N = 40; Fch = F change; R^2^ ch = Adjusted R^2^ change.

In summary, Predictors in Step 1 (Anxiety Regulation and Rumination) predicted 10% of the variance in *Stress Regulation*, but did not significantly predict *Nervous*, *Upset*, *Sadness* or *Depressed Affect Regulation*. In contrast, Step 2 predictors (Baseline NA and NA Reactivity) explained an additional 48–59% of the variance in NA Regulation, but did not significantly predict *Depressed Affect Regulation*. Higher baseline *NA* and more *NA* reactivity predicted significantly greater *NA Regulation*. The hypothesis that Anxiety Regulation scores on the AFML scale would predict greater regulation of induced NA was not supported. There was partial support for the hypothesis that Rumination would relate to less regulation of NA by listening to music.

#### Predictive effect of familiarity and music-specific efficacy beliefs

Given their predictive power in the previous regression analyses, the covariates Baseline NA and NA reactivity were entered in Step 1. Participants’ ratings of familiarity and perceived efficacy scores were entered in Step 2. Results of this analysis are presented in Table 10 and summarised below.

**Table 10:** Hierarchical multiple regression examining the influence of familiarity of music and music-specific efficacy beliefs on negative affect regulation in 20 younger adults and 20 older adults assigned to a self-chosen music listening intervention.

	Stress Regulation			Nervous Regulation			Upset Regulation			Sadness Regulation			Depressed affect Regulation		
	*F*_(4,35)_ = 23.23, *p* < .001, *R*^2^ = .73, *Adj R*^2^ = .70.			*F*_(4,35)_ = 14.43, *p* < .001, *R*^2^ = .62, *Adj R*^2^ = .58			*F*_(4,35)_ = 19.09, *p* < .001, *R*^2^ = .66, *Adj R*^2^ = .62			*F*_(4,35)_ = 14.08, *p* < .001, *R*^2^ = .62, *Adj R*^2^ = .57			*F*_(4,35)_ = 4.40, *p* < .01, *R*^2^ = .33, *Adj R*^2^ = .26		
Predictors	β	*F*ch	*R*^2^ ch	β	*F*ch	*R*^2^ ch	β	*F*ch	*R*^2^ ch	β	*F*ch	*R*^2^ ch	β	*F*ch	*R*^2^ ch
**Step 1**		37.19***	.65		25.93***	.56		23.17***	.53		18.48***	.47		2.96	.09
**(i) Baseline NA**	.29**			.52***			.09			.20			.30		
**(ii) NA Reactivity**	.83***			.91***			.75***			.78***			.36*		
**Step 2**		3.75*	.05		1.81	.02		5.40**	.09		5.34**	.10		5.17*	.17
**(i) Familiarity**	.09			.08			.20*			.20			.30*		
**(ii) Perceived Efficacy**	.23*			.19			.27*			.28**			.36*		

Notes

* p < .05

** p < .01

*** p < .001.

N = 40; Fch = F change; R^2^ ch = Adjusted R^2^ change.

Predictors in Step 1 (Baseline NA and NA Reactivity) continued to explain the greatest proportion of variance in NA *Regulation* (47% - 65%). Step 1 predictors did not significantly predict *Depressed Affect Regulation*. However, Predictors in Step 2 (Familiarity and Perceived Efficacy) accounted for additional variance in 4 of the 5 models (range: 5–17%). The hypothesis that the perceived efficacy of the music selected for the intervention would predict greater NA regulation was supported. Participants’ ratings of familiarity of the music had a less significant role on regulation of induced NA than music-specific efficacy beliefs.

## Discussion

The current RCT examined the efficacy of a brief music listening intervention for the function of affect regulation. The hypothesis that a self-chosen music listening intervention will be more effective in regulating induced NA than an experimenter-chosen active control was supported. Specifically, post-intervention the NA regulation score was higher, signifying better regulation for those in the music listening condition (intervention) than for those listening to a radio documentary (control). Correcting for multiple NA outcomes and using a more conservative p-value of .01, there was a significant main effect for condition found for *Nervousness* and *Sadness*, with all other NA outcomes excluding *Tension* showing positive effects of self-chosen music at the .05 level of significance. Overall, effect sizes were medium to large ranging from .06 –.17. Consistent with the studies by Sleigh and McElroy [21], Matsumoto [20] and Radstaak et al. [25], which found positive effects of music relative to an active control, the current study found that listening to music had a greater regulatory effect than listening to a radio documentary. More generally, this finding suggests that listening to personally-chosen music may provide an effective means of regulating affect in times of stress.

The majority of studies have induced NA prior to music listening, therefore examining recovery from NA rather than regulation of ongoing NA. For example, using the TSST Khalfa et al. [41] found that listening to relaxing music supported recovery from stress. Other designs, such as the current study, have had participants listen to music after introducing a stressor but before the completion of a stressor [19, 26]. In the current study, there was a significant increase in self-reported NA following the speech preparation and arithmetic tasks, and a significant reduction in NA following the music listening intervention. This is consistent with previous research demonstrating benefits of music on NA regulation before the cessation of a stressor [27, 42]. Only one study has examined the protective effect of music by applying the TSST after a music listening intervention, and results did not support a protective effect [36]. Additional studies are needed to understand when music is most effective for affect regulation, and the optimal timing of music interventions relative to NA induction procedures.

Regression analyses revealed that a stronger negative reaction to the induction and higher levels of NA at baseline related to greater regulation of NA after listening to music. Controlling for these variables in the ANCOVA analyses comparing music listening to an active control condition thus provides strong evidence supporting the benefits of self-chosen music listening on affect regulation. This analytical approach represents an important advance by the current study, as other studies have not controlled for reactivity, with the exception of the study by Knight and Rickard [42] which did covary participants’ baseline affect. At the same time, there are broader individual difference variables that may also impact reactivity and regulation of NA, including trait neuroticism and emotion regulation abilities, that were not controlled for in the current study and should be included in future studies of musical affect regulation.

The lack of significant interaction between age group and experimental condition in the current study suggest that younger and older adults do not respond differentially to a self-selected music listening intervention relative to an active control condition. It is noteworthy that the affect regulation benefits in younger and older adult groups were so similar, particularly given that younger and older adults did differ in the type of music they selected for coping with a stressful situation. The common affect regulation benefits observed in both younger and older adult groups in this study supports the suggestion that, even in the context of significant variation in music selections, individuals across the lifespan are competent at selecting music that is suitable for addressing their personal needs [43].

The positive effects of personally chosen music listening in the current study may be explained in part by the role of choice and control as a mechanism of stress reduction. Each participant in the intervention listened to music personally chosen for a stressful situation, whereas participants in the control condition listened to a researcher-assigned radio documentary. The regulatory effects of music may be enhanced when stimuli are self-selected due to increased feelings of control, dominance, and agency [44], which may assist with adapting to stressors [45]. The absence of choice or control in the active control condition may have impacted its relative efficacy. Having said that, the control group experienced the same advance level of control (i.e., they were also allowed to select music in advance) and the intervention group did not have any further control in the experimental session—they were offered no further choices and were simply provided with an opportunity to listen to the music that the experimenter made available from two options the participant had previously selected. Though it was not as significant, a reduction in NA was observed in the active control condition. This suggests, at least, that control is not the sole mechanism of this regulatory effect.

The vast majority of participants selected music that they themselves rated as extremely familiar. Emotional effects of music are said to be enhanced by familiarity and liking [31, 32]. In the current study, familiarity of participants’ music selections predicted some regulation effects of music, but had a minor role relative to participants’ ratings of the perceived regulation efficacy of the music they chose. Consistent with Social Cognitive Theory [39] and empirical research on effects of music listening [40] participants’ efficacy beliefs about the regulating effects of their self-chosen music predicted greater regulation of induced NA in the current study. This promising line of enquiry should be pursued, and future studies should seek to manipulate the effects of choice and control, even in the context of familiar, well-liked music listening experiences, to disentangle the independent and interactive effects of choice, efficacy beliefs, and familiarity/liking on affect regulation in stressful situations.

Music listening is a complex intervention with a number of potential mechanisms underlying its’ regulating effects. A new model of musical affect self-regulation has recently been put forward [46], drawing together the BRECVEMA framework which describes 8 mechanisms by which music listening exerts its emotional effects [47] and the *Goals*, *Strategies*, *Tactics* and *Mechanisms* (GSTM) framework [48]. Within this model music listening is viewed as one possible *Tactic* to achieve affect regulation *Goals*, using different *Strategies* (e.g., distraction, reappraisal) and through a multitude of *Mechanisms* of action. Future studies should attempt to measure more of the *Strategies*, *Tactics*, and BRECVEMA mechanisms. The current study moves in this direction by including familiarity and perceived efficacy ratings, and also by examining effects of efficacy beliefs regarding the affect regulation functions of music listening, and by comparing music listening against another potential *Tactic* for regulating NA in everyday life (i.e., listening to a radio documentary). The analysis of effects of other *Strategies*, and BRECVEMA mechanisms will require the development of additional scales that provide valid and reliable measures of these factors, and also the design of new experimental protocols that allow us to disentangle discrete mechanisms.

### The effects of music on younger and older adults

The current study also revealed a consistent significant main effect for age group on affect regulation. As hypothesised, older adults experienced greater recovery from NA than younger adults did, regardless of the tactic employed for affect regulation (music listening or listening to a radio show). One interpretation for this effect is that it reflects more general positive developmental changes that occur across the lifespan. Lower reported NA at baseline and greater affect regulation effects in older adults compared with younger adults in response to both conditions of the current study is consistent with socio-emotional selectivity theory and related empirical work demonstrating a reduction and stabilisation of NA and improved affect regulation abilities in older adulthood [9–11].

Older adult participants may have experienced significantly greater NA regulation across both experimental and control conditions because they also enjoyed the active control condition. Alternatively, this effect could be due to developmental changes in affective processing, specifically the positivity effect [12]. This positivity bias has been observed in affect identification in speech and in music listening [13]. As such, older adults may have been attuned to more positive speech features in the radio documentary relative to younger adults in the control condition. Similarly, the positive effects of self-chosen music listening observed may be driven, in part, by differences in enjoyment or liking across conditions. However, this potential difference in liking the control condition was not measured or controlled for in the current study, which makes this interpretation of effects speculative, but worthy of future investigation. It is notable that average NA Regulation scores were highest for older adults in the music listening condition. This suggests that, in addition to other sources of positivity, music listening may be a beneficial strategy for regulating stress for healthy older adults.

Findings regarding age differences, however, should be interpreted with caution, as the younger and older adult samples differed across variables other than age. They also differed in their incentives to participate. Younger adults were drawn from a convenience sample of university students, the majority of whom received course credit for their participation. Older adults were volunteers drawn from a community sample. As predicted, younger adults were more *Sad*, *Upset*, *Bored*, *Fatigued* and *Stressed* at baseline, potentially due to different incentives to participate in the study—or because of more fundamental developmental differences in affective functioning. However, by statistically controlling for group and individual differences in baseline affect in the analyses, confidence in reported findings regarding the benefits of music listening for affect regulation is increased.

### Limitations

The primary focus of this study was age differences in the regulating effects of music listening in everyday life, and the choice of a self-selected music listening intervention and an active control condition reflects that focus. However, there are additional experimental controls that could be employed to understand the nature of music listening effects more broadly. For example, a third condition contrasting the relative effects of experimenter-selected music would have been useful. More generally, while the current study compared a self-selected music listening condition with an experimenter-selected active control condition, to understand the impact of familiarity, choice and control on regulation effects it would be important to compare self-selected and experimenter-selected music with both a self-selected and experimenter-selected active control condition (i.e., four conditions). Finally, although there has been some debate as regards the use of non-active controls, and silence in particular [18], a further option would be to compare the four conditions above with a silent control condition.

Participants were self-selected and motivation to participate may have arisen from a desire to confirm the benefits of music. A number of steps were taken preemptively to minimise potential bias stemming from demand characteristics. Firstly, to avoid a direct focus on affect regulation, participants were informed that the study was concerned with the effect of music on task performance. Allocation to the control or intervention group was concealed from participants. The intervention or control condition itself was presented as a break or rest period in the experimental procedure, and participants in the control condition listened to their self-selected music after the final assessment. It is hoped that taking this approach reduced the confounding effect of demand characteristics on findings of the current study.

Following Russell [33], self-report measures of affect employed in this study adopted a bipolar definition of affect, with scales ranging from positive to negative affective experience—rather than bivariate, where positive and negative affect are rated independently. Independent ratings of positive and negative affective experience may have provided further information. Furthermore, mixed and complex emotions, including the co-experience of positive and negative emotions, are more common in aesthetic experience [47]. These mixed and complex emotions could not be examined using the VAS measure of affect adopted here.

Finally, this study does not address how music was selected. Music listening can be informed by the function of music listening being served [49] and the music listening context [50]. While participants in the current study selected music with affect regulation functions in mind, it is unclear how participants approached the selection process. Studies that examine the process of selecting and listening to music in real-world stressful situations are needed to evaluate the specific selection processes used, and extent to which findings from the current laboratory study generalise to real-world situations.

## Conclusions

The results of this study provide preliminary insights into the effects of self-chosen music on NA amongst younger and older adults, and supports the idea that, relative to listening to an experimenter-chosen radio documentary, personal music listening offers additional advantages for regulating NA aroused by the prospect of a stressful challenge, and this effect does not differ for younger and older adults. Future research employing additional experimental controls is needed to examine the mechanism through which self-selected music listening serves to regulate NA in stressful situations. Supporting the generalisability of the socio-emotional selectivity theory, older adults showed superior affect regulation than younger adults in both music listening and active control conditions.

## Supporting information

S1 File Dataset. (SAV)
